# Histopathology of livers in patients with congenital portosystemic shunts (Abernethy malformation): a case series of 22 patients

**DOI:** 10.1007/s00428-018-2464-4

**Published:** 2018-10-24

**Authors:** Claudio De Vito, Athanasios Tyraskis, Mark Davenport, Richard Thompson, Nigel Heaton, Alberto Quaglia

**Affiliations:** 1 King’s College Hospital, Institute of Liver Studies, London, UK; 2 Division of Clinical Pathology, Geneva University Hospital, Geneva, Switzerland; 3 Department of Paediatric Surgery, King’s College Hospital, London, UK; 4 Paediatric Liver, GI &amp; Nutrition Centre, King’s College Hospital London, London, UK; 5 Department of Cellular Pathology Royal Free Hospital, University College, London, UK

**Keywords:** Porto-systemic shunt, Liver, Abernethy malformation, Nuclear vacuolation

## Abstract

Congenital portosystemic shunt (CPSS) is a congenital anomaly resulting in partial or complete diversion of the portal blood into the systemic circulation. The literature on the histological changes in livers of patients with CPSS is limited. Liver histology of 22 consecutive patients managed in our institution between 2001 and 2016 was reviewed. Twenty-one patients were children at the time of diagnosis. Thirty-two specimens were available and consisted of three explant livers and 29 biopsy samples from 19 patients. Sixteen samples were from wedge biopsies taken at the time of shunt closure. Thirteen were from core needle biopsies taken during clinical work-up. A variable proportion of portal tracts contained prominent thin-walled channels (PTWCs) and arterio-biliary dyads. The proportion of portal tracts containing triads, arterio-biliary dyads and biliary monads varied considerably in the different samples. Dilated inlet venules, increase in the number of portal arteries or the presence of portal arteries of increased size, deposition of copper-associated protein, sinusoidal dilatation, capillarization and intralobular individual arteries were present. Physiological nuclear vacuolation of periportal hepatocytes was absent in most samples from our paediatric patients. Presence of PTWCs, arterial-biliary dyads, increased arterial profiles in portal tracts and lobule and lack of the physiological periportal vacuolated hepatocytes in children are the most characteristic histological changes of CPSS in the liver periphery.

**Electronic supplementary material:**

The online version of this article (10.1007/s00428-018-2464-4) contains supplementary material, which is available to authorized users.

## Introduction

Congenital portosystemic shunt (CPSS) are congenital anomalies resulting in partial or complete diversion of the portal blood into the systemic circulation. First described by John Abernethy [1], they are thought to be related to defective portal vein embryogenesis, as discussed by Wanless et al. [2].

CPSS are increasingly being diagnosed in the neonatal period when an ultrasound is being performed for neonatal jaundice. Another large proportion is picked up incidentally when having an ultrasound for unrelated reasons. The remainder are usually identified due to the associated complications of neurocognitive dysfunction, developmental delay, hepatocellular nodules and/or hepatopulmonary syndrome. If gone unnoticed until adult life, presentation may be due to symptoms related to a hepatic nodule, signs of low-grade encephalopathy, pulmonary hypertension, hypoglycaemia, chronic abdominal pain and/or elevated ammonia levels.

The literature on the histological changes in livers of patients with CPSS is limited. Wanless et al. [2] were the first to describe the histological findings and in particular portal vein hypoplasia and large regenerative nodules in the liver of a patient with persistent ductus venosus. In addition to other individual case reports [3–7], the case series by Lisovsky and colleagues [8] on five CPSS patients, to our knowledge, is the most comprehensive and detailed histological description to date. We describe, therefore, our findings in a larger series of 22 patients with CPSS who were managed clinically in our institution over a period of 15 years, and from whom background liver tissue was available for histological review. The emphasis in this paper is on the hepatic histological changes related to the effect of the underlying anomaly rather than the more general clinical aspects or details of the liver tumours identified in some of these patients.

## Material and methods

Our case series consists of 22 of 46 consecutive patients diagnosed and managed in our institution for CPSS between 2001 and 2016 for which liver specimens were available (core needle biopsy, wedge biopsy or liver explant).

The type of CPSS was subclassified into four types:

- extrahepatic end to side shunt with no detectible flow into the intrahepatic portal system, (type 1);
- extrahepatic either side to side or H type shunt with some preserved intrahepatic portal flow, (type 2);
- intrahepatic shunt(s), any configuration except for persistent ductus venosus (intrahepatic type);
- persistent ductus venosus (PDV) [9–13]

We defined PDV according to the criteria by Blanc et al. [12]: ‘intrahepatic shunt running from the proximal part of the left portal branch to the terminal part of the left hepatic vein and located in the depth of the Arantius sulcus between the left and caudate lobes of the liver’. We considered as intrahepatic shunts any intrahepatic shunts that did not fulfil Blanc’s criteria for PDV.

The histology review was carried out by AQ and CDV and the following features were assessed: Fibrosis was assessed in a descriptive semiquantitative fashion noting its location (portal or centrilobular/perisinusoidal based), and whether it was associated with septa and parenchymal nodular transformation. This assessment was carried out with the aid of the Gordon and Sweet reticulin and Sirius Red stains carried out routinely in our laboratory. Particular attention was paid to changes to portal vascular structures. The term ‘hypoplasia’ was used for portal vein branches showing a thickened wall and/or abnormally small calibre relative to the size of the nearby bile ducts and/or arteries, in line with the terminology used by Wanless et al. [2] and Lisovsky et al. [8]. Portal tracts containing one or more arterial profiles and at least one interlobular or septal bile duct and without an obvious portal vein branch, or with one or more slit-like spaces which needed immunohistochemistry to be characterised further were recorded as ‘dyads’. Portal tracts containing a bile duct, and an artery of matching size and a larger normal-sized portal vein branch with a thin wall was recorded as a ‘triad’ [14]. Individual portal structures were recorded as monads and the type specified. Unless individual arteries had a significant cuff of stroma around them they would be recorded separately as interlobular individual arteries rather than portal arterial monads. Portal tracts containing prominent, sometimes dilated, thin-walled channels (PTWCs), which needed immunohistochemistry (podoplanin, CD34) to be characterised further, were not recorded as dyads but counted separately. Dilated thin-walled channels protruding through the limiting plate were recorded as dilated inlet venules. Portal arterial branches were considered abnormal if appeared increased in number against the reference standard described by Crawford et al. [14]. Small thick-walled capillaries were considered to be minute arterial branches. In line with the findings by Lisovsky et al. [8], the presence of abnormal arteries, much larger than the nearby accompanying bile duct was also recorded. The presence of isolated intralobular arterioles and increased sinusoidal stain for CD34 was also recorded when applicable. Additional features assessed were changes to bile ducts (bile duct loss, bile duct attenuation and peribiliary lymphocytosis) and hepatocytes (normal hepatic plates, hepatic plate disarray, nodular regenerative hyperplasia, aberrant stain for CK7, periportal accumulation of copper-binding protein, periportal hepatocyte nuclear vacuolation and steatosis).

We used a chi-squared test to investigate whether there was an association between the histological changes and the type of shunting, dividing our patients in two groups, one with type 1 shunting (complete absence of portal flow) and the other group with type 2, intrahepatic shunts of persistent ductus venosus, and whether there was an association between the histological changes and the presence or absence of liver tumours.

## Results

Our cohort consisted of 22 patients (ten females). Most were children at presentation (age range = 2 months–18 years; median 5.5 years). The only patient presenting in adulthood was a 49-year-old gentleman who was investigated for symptoms related to a large hepatocellular carcinoma. Radiological signs of an underlying CPSS were discovered during his work-up.

In terms of shunt type, six patients had a type 1, 13 had a type 2, two had an intrahepatic type and one had a persistent ductus venosus. Of the two intrahepatic shunts, one (patient 14) run from the left portal vein to the middle hepatic vein, whilst the other (patient 16) run from the left portal vein through the parenchyma of the liver to a peripheral subcapsular vessel away (to the left) from the Arantius sulcus, and subsequently fed into the left hepatic vein. The shunt type was characterised by cross-sectional imaging and portal venogram when present and then correlated with surgical findings.

Thirty-two samples in total were available for review. In three patients, the samples examined were obtained from whole livers removed at transplantation. In the other 19 patients, the samples reviewed were 16 wedge biopsies obtained at laparotomy for shunt closure and ten percutaneous core needle biopsies. Of the ten percutaneous liver biopsies, four were obtained from four children at their first clinical presentation before CPSS was discovered. One patient was a 2-month-old baby girl who presented with conjugated hyperbilirubinaemia, and the other three were older children with abnormal liver biochemistry or hepatosplenomegaly. The other six core needle biopsies were taken during surgery or to complement the clinical work-up of known CPSS. The details are shown in Table S1.

## Explanted livers

The histological findings in the three explanted livers are summarised in Table 1. All three explant specimens had multiple nodular lesions with a histological appearance ranging from focal nodular hyperplasia (FNH) to hepatocellular adenoma (HCA), well-differentiated hepatocellular neoplasm (WDHN) [15–17] or hepatocellular carcinoma (HCC) in two, and a single large HCC with marked atrophy of the remaining parenchyma in one.

**Table 1:** Explanted livers

Patient (age/sex)	Type of CPSS	Macro	Fibrosis	PV at the hilum	Small PV	Dilated thin-walled vessels	Dilated inlet venules	Portal arteries	Sinusoidal dilatation	Sinusoidal capillarisation	Individual arteries	Hepatic plates	Bile ducts	Copper-associated protein	CK7 + ve hepatocytes	Periportal vacuoles	Tumour
1 12Y/F	PDV	Multiple nodular lesions	Variable fibrosis, ranging from minimal to severe	Present (ltd)	Normal, many portal tracts with dyads only, thickened wall in places	Present, focal	Present	Present hyperplastic in places. Focal increase in size	Present	Absent	Present	Disarray. No NRH	Present	Absent	Yes, in areas of fibrosis, periportal and centrilobular	Absent	FNH/HCC
2 7Y/M	1	Large 140 mm left lobe lesion with atrophic right lobe	Severe fibrosis and marked parenchymal atrophy	Present (ltd)	NA	NA	NA	NA	NA	NA	NA	NA	NA	Present	Present	Absent	HCC
3 18Y/M	1	Multiple nodular lesions	Minimal fibrosis and patchy parenchymal atrophy	Present (ltd)	Normal, many portal tracts with dyads only, thickened wall in places	Present, focal	Absent	Present hyperplastic in places. Focal increase in size.	Present	Focal	Present	Disarray. NHR present	Present. Mild cholangitis	Focal	Absent	Absent	FNH/HCA/HCC (Sorkin et al.)

*Ltd* limited sampling, retrospective study; NA not applicable

The samples of background liver were limited as they were perilesional in two specimens (patients 1 and 2), and we cannot exclude that at least some of the changes noted were the result of tumour compression. Particularly in patient 2, there was marked parenchymal atrophy, severe fibrosis and parenchymal micronodular transformation, making this case not comparable to the other ones. Of note, the portal vein was present in the sections from the porta hepatis in all three specimens, but there were no representative systematic samples from the large portal tracts to comment further on the status of the large intrahepatic portal vein branches.

**Fig. 1:**
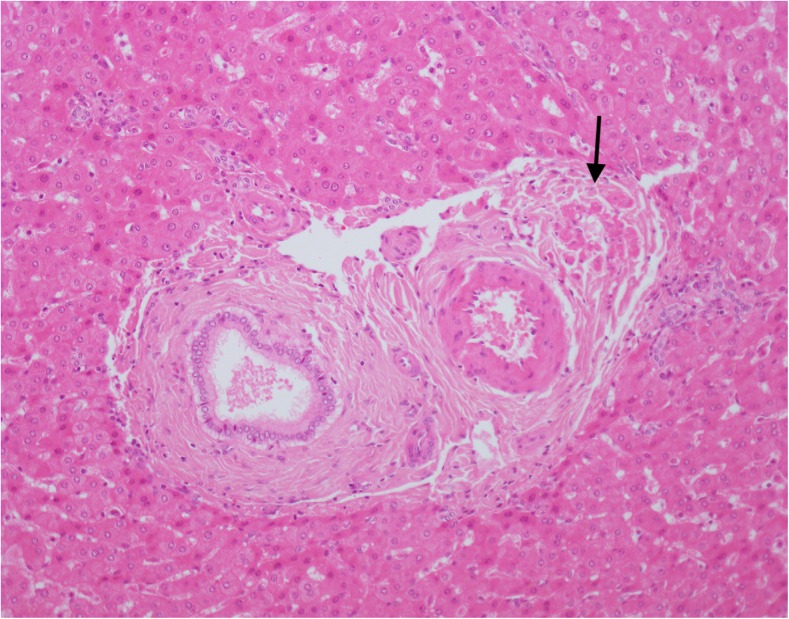
Patient 3. Hypoplastic portal vein (arrow) of small calibre, narrow lumen and thickened wall. H&amp;E. Magnification × 200

The amount of fibrosis in patient 1 was variable ranging from severe bridging fibrosis in the vicinity of the HCC to minimal portal fibrosis in the vicinity of the lesions with features of focal nodular hyperplasia. The small portal tracts in the less fibrotic areas showed some hypoplastic portal vein branches (Fig. 1) and some PTWCs in places. Some hepatic artery branches were hyperplastic or enlarged. Bile ducts were intact and cholangitis was present in one patient. There was some sinusoidal dilatation and isolated hepatic arteries were identified in the lobules.

The amount of fibrosis in patient 3 was minimal and portal-based in two samples from the right and left lobe, which had been taken as far away as possible from any of the nodular lesions. There was a patchy parenchymal atrophy with irregular spacing of the portal tracts including crowding and convergence [8] in places. Small intrahepatic portal vein branches were absent or hypoplastic in most portal tracts. Dilated inlet venules were not identified. PTWCs and arterial branches increased in size or in number were present in places. There was a hepatic plate disarray, and areas of nodular regenerative hyperplasia were also noted. Sinusoids were dilated in some of the samples, and their endothelium stained focally for CD34 often as an enhancement of the normal periportal stain as observed in the biopsy specimens. Individual lobular arteries were also observed in places.

Cytokeratin 7 positive hepatocytes and periportal deposits of copper-binding protein were present in two patients. Of note, periportal hepatocytes with vacuolated nuclei in the periportal regions were not identified in any of these three patients.

## Biopsy specimens before or at the time of shunt closure

Twenty-nine biopsy samples from 19 patients were available for review. Sixteen samples were from wedge biopsies and the other 13 from core needle biopsies. Three core needle biopsy specimens from patients 13, 17 and 22, in addition to those described in Table S1 were excluded from histological review because they were too small.

Ten patients underwent a single biopsy of background liver (five core needle biopsies and five wedge biopsies). Nine patients underwent multiple background liver biopsies. Three patients (4, 7 and 9) underwent wedge liver biopsies from both right and left lobes at the time of the shunt closure. The other six patients underwent two or more background liver biopsies at different time points. The details are described in Table 2.

The ten core needle biopsies, which were reviewed further, ranged in size from 7 mm to 35 mm (average 14.1 mm) in length and included a range of 2 to 11 complete portal tracts (median = 3.5). In contrast, the wedge biopsy specimens included a range of 5 to 71 portal tracts (median = 17). An overall summary of the histological findings in all biopsies is provided in Table S1.

## Histological findings in 16 wedge biopsies and ten core needle biopsies

Our findings are summarised in Table S1 (individual patients) and Table 2 (comparison between wedge and core needle biopsies).

**Table 2:** Histological findings between wedge and core needle biopsies

	Wedge biopsy (*n* = 16)	Core needle biopsy (*n* = 10)
Number of portal tract (median)	5–71 (17)	2–11 (3.5)
Architecture	Preserved	Preserved
Fibrosis: none; periportal/perisinusoidal; bridging	4 (25%); 5 (31%); 7 (44%)	2 (20%); 7 (70%); 1(10%)
Portal vascular structure:		
- PTWC	15 (93.8%)	5 (50%)
- Triads	8 (50%)	4 (40%)
- Dyads	15 (93.8%)	9 (90%)
- Monads	6 (37.5%)	3 (30%)
- Dilated inlet venules	7 (43.8%)	3 (30%)
- Increase in the number of arteries	14 (87.5%)	6 (60%)
- Arteries of increased size	5 (31.3%)	0
Sinusoidal dilatation (%)	10 (62.5%)	7 (70%)
Sinusoidal capillarisation	11 (68.7%)	5 (50%)
Individual arteries	10 (62.5%)	5 (50%)
Steatosis	4; mild (2) and moderate (2)	1; moderate
Deposition of copper-associated protein	7 (43.7%)	4 (40%)
Periportal vacuolated nuclei absent; very focal; present	11 (68.7%); 3 (18.8%); 2 (12.5%)	7 (70%); 1 (10); 1 (10%)*
Bile ducts**		
- Normal	11 (68.7%)	9 (90%)
- Peribiliary lymphocytosis	5 (31.3%)	0
CK7 expression in hepatocytes	3 (23.1%)***	2 (22.2%)****
Hepatic plates		
- Disarray	15 (93.8)	10 (100%)
- NRH	0	0

*The adult patient was not included since periportal vacuolated nuclei are physiologically not present in adult

**No bile duct was identified in one. This core needle biopsy (patient 12) was characterised by a considerable number of individual arteries and just two dyads but no other identifiable portal structures despite being 35 mm long. It is entirely possible that this non-targeted biopsy hit a region of regenerative hyperplasia or a large regenerative nodule devoid of proper portal structures

***Could not be assessed in three patients

****Could not be assessed in one patient

### Overall architecture and fibrosis

The overall lobular architecture was preserved in all samples. Fibrosis ranged from none to mild periportal and/or perisinusoidal but with no bridging to bridging fibrosis in the form of patchy slender incomplete or mostly porto-portal fibrous septa but without enclosure of parenchymal nodules.

### Portal vascular structures

**Fig 2:**
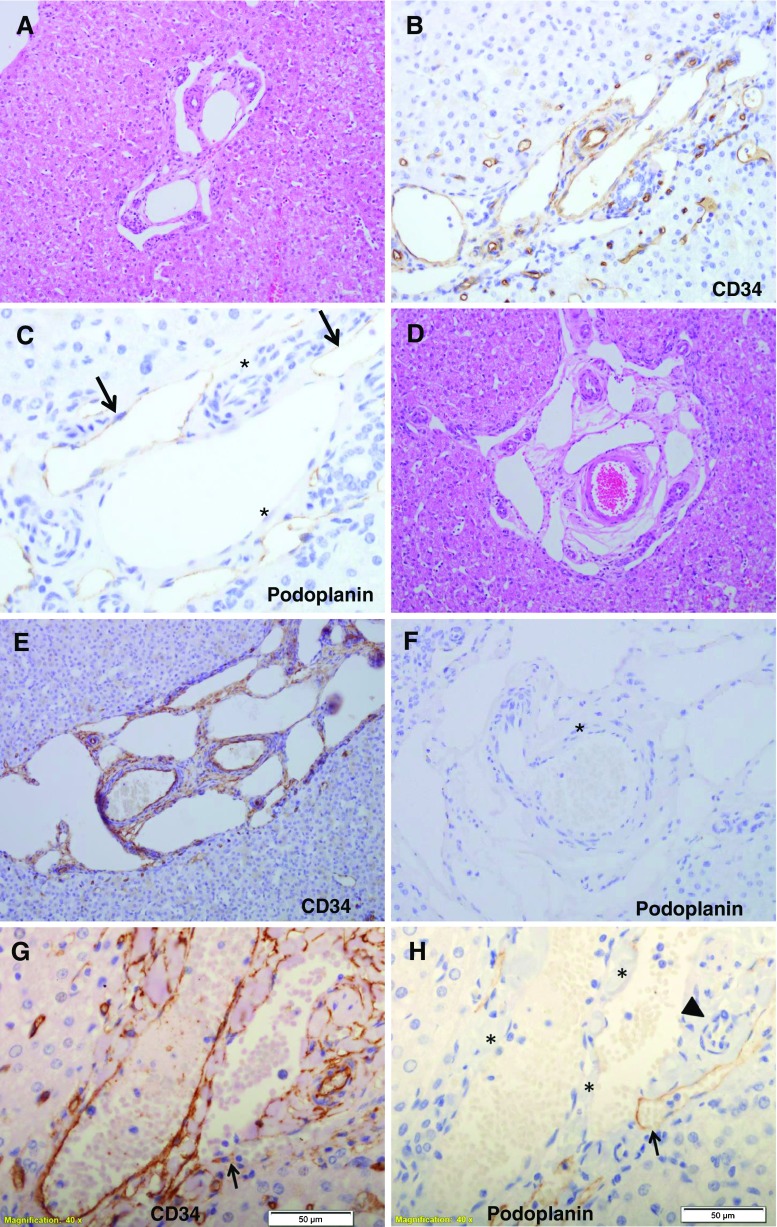
Two examples (**A**, **B**, **C** and **D**, **E**, **F**) of prominent thin-walled channels (PTWCs) in two portal tracts from patient 13. The endothelium lining these channels stain for CD34 (**B**) or for podoplanin (**C**, arrows indicate podoplanin stain, asterisks indicate the podoplanin negative endothelium of a portal arterial branch and of a portal vein branch respectively) indicating a combination of portal vein branches and lymphatics, or for CD34 only (**E**, **F**, the asterisk in **F** indicates the podoplanin negative endothelium of a portal arterial branch). CD34 (**G**, arrow) and podoplanin stain (**H**, arrow same point as arrow in **G**, indicating the podoplanin positive vessel; the triangle indicates the podoplanin negative endothelium of a portal arterial branch, and the asterisks indicate the podoplanin negative endothelium of portal vein branch) in a portal tract from patient 4 suggests porto-lymphatic shunting (arrowhead) (**G**, **H**). Original magnification: **A**, **D**, **E**, **F**, × 200; **B**, **C**, **G**, **H**, × 400. Please note that pictures **C**, **F**, **G** and **H** are shown at an enhanced magnification in order to show the details of the podoplanin stain. Panel pairs **B** and **C**, **E** and **F** and **G** and **H** each illustrate one portal tract at different levels of section

The majority of samples had portal tracts with PTWCs (Fig. 2A–D). The proportion of portal tracts showing this change varied considerably between the different samples (range = 7.7%–90.9%; median = 46.7%) and (range 16.7%–66.7%, median 8.3%) in wedge and core needle biopsy respectively. These channels in many instances were dilated, and were often wrapped around the arterial branch. Immunohistochemistry for CD34 (Fig. 2B, E) and podoplanin (Fig. 2C, F) was available in all samples except one and showed that these channels corresponded to portal vein branches or a combination of lymphatics and portal vein branches. We found it difficult to draw a clear separation between these two structures in many instances. Lymphatics and portal vein branches were often in very close proximity and in one portal tract of one sample from patient 4, there appeared to be communicating, suggesting porto-lymphatic shunting (Fig. 2G, H).

**Fig. 3:**
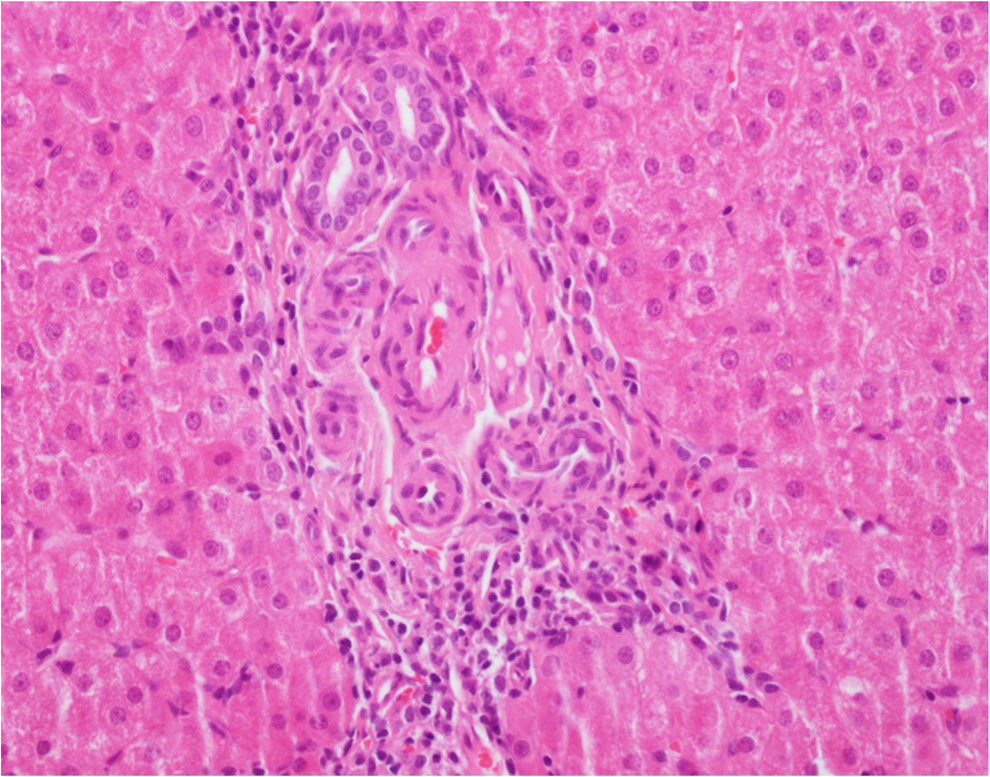
Patient 10. Portal tract including an interlobular bile duct and arterial branch of matching size but no portal vein. Narrow slit-like spaces could be of portal derivation. Please note also increase number of arterial profiles and the absence of vacuolate nuclei in periportal hepatocytes. H&amp;E. Magnification × 400

**Fig. 4:**
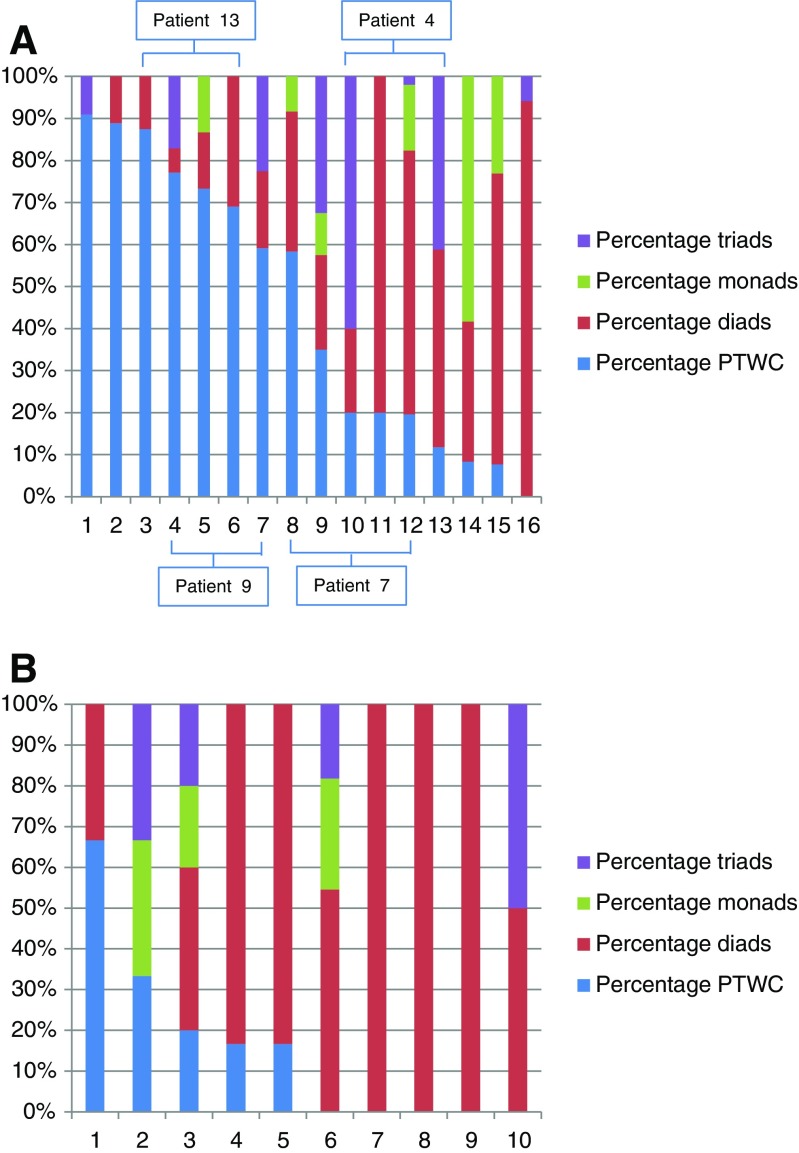
Proportion of PTWCs, triads, dyads and monads in wedge (A) and core needle (B) biopsies

**Fig. 5:**
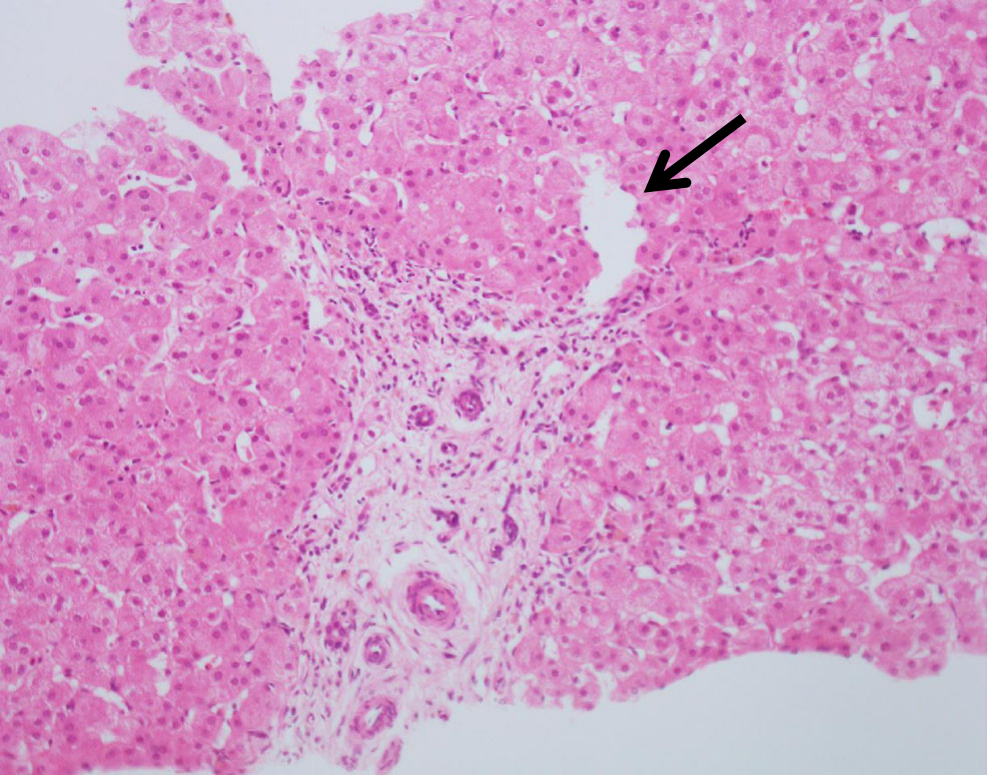
Patient 16. A dilated inlet venule (arrow) protrudes through the limiting plate. H&amp;E. Magnification × 200

**Fig. 6:**
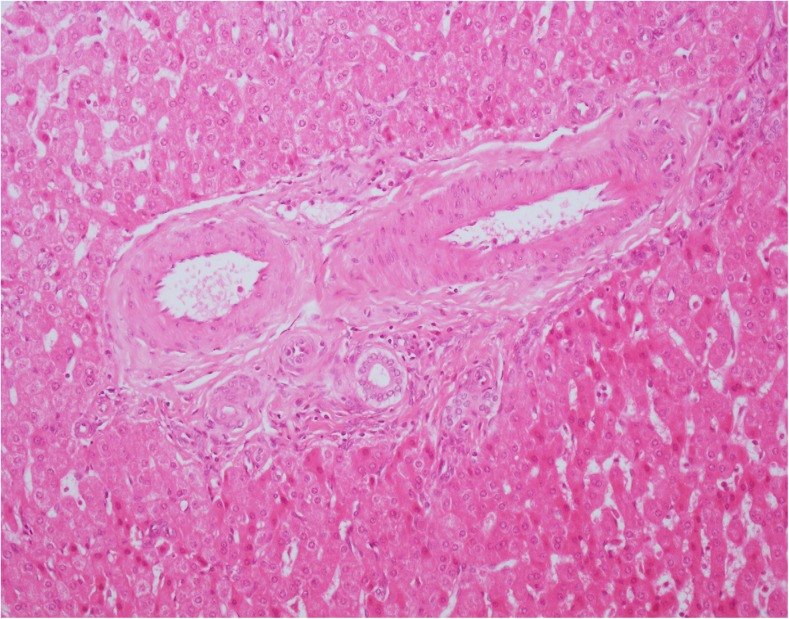
Patient 3: H&amp;E section shows an artery branch of much larger size than the nearby bile duct (magnification × 200). Please note also increase number of arterial profiles and the absence of vacuolate nuclei in periportal hepatocytes

Portal vein hypoplasia and the presence of portal arterio-biliary dyads and biliary monads were therefore assessed in the portal tracts in which PTWCs were not present. We were not able to identify hypoplastic portal veins (i.e., of an unusual small size compared to the other portal tract structures and without a thickened wall). Some candidate portal hypoplastic vein branches were considered in places, but we were left with the doubt that their small size was due their partial representation in the plane of section examined. A portal vein branch with a thickened wall was spotted in one portal tract only (patient 8). We, therefore, counted the proportion of portal tracts without PTWCs containing triads, dyads and monads (Fig. 3). Their relationships with other portal vascular changes are shown in Fig. 4 and Table 3. Similarly to PTWC, the proportion of portal tracts containing triads, dyads and monads varied considerably between the different samples (triads: range 0–60%, median = 1%; dyads: range = 0–94%, median = 26.7%; monads: range 0–58.3% median 0%) in wedge biopsy. The proportion of portal tracts containing triads, dyads and monads in core needle biopsy also varied considerably (triads: range 0–50%, median = 0%; dyads: range = 0–100%, median = 68.9%; monads: range 0–33% median 0%). Not surprisingly, they were more often observed in those samples with a lower proportion of portal tracts containing PTWCs (Fig. 4). In wedge biopsy, 15 of 16 samples showed a combination of PTWCs and dyads. In the single sample in which PTWCs were not observed, more than 90% of portal tracts contained dyads. Of note, dilated inlet venules (Fig. 5), increase in the number of arteries or the presence of arteries of increased size (Fig. 6) were present in variable proportion between wedge and core needle biopsies (Table 2).

**Table 3:** Portal changes (portal thin-walled channels (PTWC), dilated inlet venules, increased number in portal arteries, increased size of portal arteries), in wedge (A) and core needle (B) biopsies. The samples are sorted according to the proportion of PTWC in a decreasing order

Patient	PTWC (%)	Dyads (%)	Monads (%)	Triads (%)	Dilated inlet venules	Increase in number of arteries	Increase in size of arteries
**A**							
19	90.9	0.0	0.0	9.1	Absent	Present	Present
17	88.9	11.1	0.0	0.0	Present	Present	Absent
13	87.5	12.5	0.0	0.0	Present	Present	Present
9	77.1	5.7	0.0	17.1	Present	Present	Present
22	73.3	13.3	13.3	0.0	Absent	Present	Absent
13	69.0	31.0	0.0	0.0	Absent	Present	Absent
9	59.2	18.3	0.0	22.5	Present	Present	Present
7	58.3	33.3	8.3	0.0	Absent	Present	Present
8	35.0	22.5	10.0	32.5	Present	Present	Absent
4	20.0	20.0	0.0	60.0	Present	Present	Absent
6	20.0	80.0	0.0	0.0	Absent	Absent	Absent
7	19.6	62.7	15.7	2.0	Absent	Present	Present
4	11.8	47.1	0.0	41.2	Present	Present	Absent
21	8.3	33.3	58.3	0.0	Absent	Absent	Absent
18	7.7	69.2	23.1	0.0	Absent	Present	Absent
10	0.0	94.1	0.0	5.9	Absent	Present	Absent
**B**							
5	66.7	33.3	0.0	0.0	Absent	Present	Absent
19	33.3	0.0	33.3	33.3	Absent	Absent	Absent
20	20.0	40.0	20.0	20.0	Absent	Present	Absent
11	16.7	83.3	0.0	0.0	Present	Absent	Absent
15	16.7	83.3	0.0	0.0	Absent	Present	Absent
5	0.0	54.5	27.3	18.2	Absent	Absent	Absent
12	0.0	100.0	0.0	0.0	Absent	Present	Absent
14	0.0	100.0	0.0	0.0	Absent	Present	Absent
15	0.0	100.0	0.0	0.0	Present	Absent	Absent
16	0.0	50.0	0.0	50.0	Present	Present	Absent

### Other features

Sinusoids were dilated in most samples (range 62.5%–70%). This change was usually patchy and did not show a preferential location (i.e., no particular predominant periportal midlobular or perivenular distribution). Increased sinusoidal endothelial stain for CD34 was present (range 50%–68.7%) but was focal, often as an enhancement of the normal periportal stain. Intralobular individual arteries were present in 15 samples (50%–62%). Hepatic plates showed a variable degree of disarray but nodular regenerative hyperplasia was not observed.

Steatosis was present in five samples, mild in two samples and moderate in the other three. Of note, one of the samples with mild steatosis was from the left lobe of the liver of patient 4 who had a concomitant right lobe biopsy, which did not show any steatosis. Deposits of copper-binding protein were present in periportal hepatocytes in 11 patients. In five other samples, it was regarded as physiological in view of the patient’s age at the time of the biopsy (three and two samples in wedge and core needle biopsies respectively). Cytokeratin 7-positive hepatocytes were observed in five samples only, and could not be assessed in four samples. Of note, 18 samples (68.7%–70%) from young patients (in whom usually this change is considered to be physiological) did not show vacuolated nuclei in periportal hepatocytes. These were present in seven samples from other young patients, although in four of them they were very focal. Bile ducts were normal in 20 samples (68.7%–90%). There was a mild peribiliary lymphocytosis in five samples (30.6%).

In summary, all samples showed abnormal portal vascular structures, mostly in the form of a variable combination of PTWCs, arterio-biliary dyads and increase in the number of portal arteries. The overall changes in the core needle biopsies were similar to those in the wedge biopsies with perhaps a lower proportion of biopsies showing PTWCs and a higher proportion of dyads. We believe that this difference could be simply due to the much smaller size of the core needle biopsies.

### Comparisons in patients with multiple samples

Some of our patients had biopsies taken from both lobes, and other biopsies taken at a different time point, allowing us to investigate whether changes varied considerably between different areas of the liver or over time.

Patients 4, 7 and 9 had wedge biopsies taken from both right and left lobes at the time of the shunt closure; we did not notice particular differences when the pair biopsies were compared, particularly in relation to the portal vascular changes. Figure 4 shows that the paired biopsies tended to cluster in terms of the proportion of PTWCs, triads, dyads and monads. One exception was the presence of mild steatosis in the sample from the left lobe compared to no steatosis in the one from the right lobe in patient 4.

Patients 13, 14, 15 and 19 had multiple samples taken at different time points. Patient 13 had two wedge biopsies at 3 years (shunt closure) and 5 years (repeat shunt closure) and which showed similar changes. In the other patients, the different biopsies were either a combination of core needle and wedge biopsies or needle biopsies only, and we believe that any difference could be due to sampling given their small size, and are not sufficient to make assumptions on how the histological features could change over time.

### Liver tumours

Seven patients had a concomitant hepatocellular lesion in the liver parenchyma, with HCC diagnosed in three patients and FNH in the other five (see Table S1).

## Correlation with type of shunting or presence of tumours

We did not find any significant statistical difference (not shown) in histological changes when comparing the wedge biopsies of two patients with type 1 shunt with those of ten patients with type 2 shunt, or the wedge biopsies of two patients with tumours with those of ten patients without. We did not include the core needle biopsies and the explanted livers because they were not comparable to the wedge biopsies, due their much smaller size (core needle biopsies), and relatively limited amount of non-lesional tissue (explanted livers), as mentioned earlier.

## Discussion

The literature on the spectrum of histological changes in livers with patients with CPSS is limited to case reports [2–7] and the single case series by Lisovsky and colleagues [8]. Our larger cohort has allowed us to confirm broadly on previous observations and add some new findings. Compared to biopsies, surgical specimens should have the advantage of providing more tissue for histological examination. In our cases, however, all resection specimens contained tumours, and the amount of non-neoplastic tissue available for this retrospective review was limited and mainly perilesional. We cannot therefore exclude that the changes present in these specimens were due, at least in part, to the effect of tumour compression.

In contrast to Lisovski et al. [8], we could identify the portal vein in the porta hepatis of all three liver explants. In patient 1, the shunt consisted of a persistent ductus venosus suggesting that it formed in the context of a developed intrahepatic portal vein tree. Patients 2 and 3 had a type one shunt characterised by an end to side connection between the extrahepatic portal vasculature and the inferior vena cava with complete lack of intrahepatic portal flow. The close and complex developmental relationships between the prerenal section of the inferior vena cava and the plexiform subdiaphragmatic anastomosis and remodelling of the vitelline veins from which the portal vein derive [18], could explain the paradoxical coexistence of a complete shunt and the hilar portal vein.

In most of our wedge biopsy specimens, portal arterio-biliary dyads were observed frequently. They do not necessarily indicate a pathological status, as they are present in up to 38% of portal tracts in liver biopsies from adult normal liver [14]. In many of our cases, a slit-like space was present in the vicinity of a dyad but it was not possible to be certain whether it represented a portal vein remnant. We do not use routinely trichrome stains, which may be helpful in evaluating portal structures and scarred vein remnants [8], and, as mentioned earlier, some of these small structures tend not to be represented in sections at different levels. We believe, however, that a threshold of 38% of portal tracts with dyads as a physiological finding does not apply to paediatric livers with CPSS because of the presence of the other vascular abnormalities in most portal tracts. The presence therefore in a biopsy specimen of a combination of dyads, biliary monads, PTWCs, dilated inlet venules and increased number of portal arteries should suggest the presence of a CPSS. PTWCs in particular were a frequent finding and relatively easy to identify. They appeared to be composed of a combination of portal vein branches and lymphatics, in some instances, and portal vein branches only in others, but this should be explored in more details by using serial sections, double epitope immunohistochemistry and, ideally, 3D reconstruction. The application of cognitive algorithms could also be informative [19, 20]. Such an approach could also be helpful to clarify whether the presence of porto-lymphatic shunting suggested in one of the biopsies does really occur. As suggested by Kanazawa et al. [10], the configuration of the microscopic portal circulation could help in predicting intrahepatic portal flow after shunt occlusion or plan for a two-stage approach. This consists of initial shunt reduction to allow the portal network to adapt to the increase in portal inflow, followed by complete occlusion a few months later [21].

Vacuolation of the nuclei of periportal hepatocytes is routinely observed in livers from young patients [22]. The absence or marked reduction of this change in most of the young patients in our series is intriguing. Commonly observed in pathological conditions such as diabetes mellitus and Wilson disease, Aravinthan et al. [23] suggested that it may be an indication of hepatocyte senescence. Serial liver biopsies from depancreatised baboons [24] showed that periportal hepatocytes develop nuclear vacuolation within 4 days, and that by 8 days almost all hepatocytes in the lobule show vacuolated nuclei. This change is reduced, but not eliminated, by the administration of insulin. Starzl et al. [25] showed in an animal model of partial portocaval transposition that exposure of the liver parenchyma to caval blood or blood of small intestinal origin results in atrophy. In contrast, the splanchnic blood returning from the pancreas, proximal part of the duodenum, stomach and spleen leads to hypertrophy and hyperplasia. In terms of hepatotropic factors, Starzl et al. [25] concluded that insulin is the dominant anabolic hormone, counterbalanced to some extent by glucagon and epinephrine. The lack of periportal hepatocyte nuclear vacuolation in our paediatric patients is therefore a paradox as one would expect the lack or reduced portal circulation to be detrimental by reducing the delivery of insulin to hepatocytes. An alternative explanation resides in the concept that nuclear vacuolation could be an indication of hepatocyte senescence, and that senescence is part of liver remodelling, including during embryonal development [26–30]. Nuclear vacuolation in children, therefore, could be an indirect sign of liver growth in the context of a preserved portal circulation or, in a broader sense, a balanced artero-portal perfusion. Unbalanced porto-arterial perfusion due to the presence of a CPSS could alter senescence and remodelling and ultimately the growth process. Whether perturbation of the senescence process influences the development of tumours in these patients is a possibility.

The presence of biliary changes and signs of chronic cholestasis in some of our patients is similar to the findings of Lisovski et al. [8] but their cause remains uncertain.

In conclusions, our series of patient with CPSS has shown a constellation of changes in the liver parenchyma, which are probably the combined effect of the underlying developmental abnormality and compensatory effects. In our experience, the most characteristic findings in the peripheral liver parenchyma include the presence of portal prominent thin-walled channels, arterial-biliary dyads, increased arterial profiles in the portal tracts and the lobule and frequent lack of the physiological periportal-vacuolated hepatocytes in children. These changes may not be fully represented in core needle biopsy samples due to sampling variation. Whether and how these changes provide the background to the development of hepatocellular tumours in these patients remain uncertain. Patients with CPSS should be considered to be at risk of developing hepatocellular tumours with the potential to progress to HCC and should be managed accordingly.

## Electronic supplementary material

ESM Table 2 (DOCX 34.9 KB)
