# Supplementary material for: Histopathology of livers in patients with congenital portosystemic shunts (Abernethy malformation): a case series of 22 patients
Source: Virchows Arch. 2018 Oct 24;474(1):47–57. doi: 10.1007/s00428-018-2464-4 (PMC6323085; doi:10.1007/s00428-018-2464-4)
Supplement: Supplementary file 1 — (DOCX 34.9 KB) [file 428_2018_2464_MOESM1_ESM.docx]

Table 2. Summary of histological findings in wedge and core needle biopsy specimens.

| **Patient** | **Sex/Age** | **Type of shunt** | **Time of biopsy** | **Fibrosis** | **PTWC**  **(PTWC /overall portal tract)** | **Portal triads/diads and monads** | **Dilated inlet venules** | **Portal arteries**  **Number/size** | **Sinusoidal dilatation** | **Sinusoidal capillarisation** | **Individual arteries** | **NRH** | **Steatosis** | **Copper associated protein** | **CK7+ve hepatocytes** | **Periportal vacuoles** | **Bile ducts** | **Tumour** |
| --- | --- | --- | --- | --- | --- | --- | --- | --- | --- | --- | --- | --- | --- | --- | --- | --- | --- | --- |
| **4** | Male/  2 years | 1 | At shunt closure, left lobe wedge | Slender bridging | Present (2/10) | 6/2/0 | Present | Increased/Normal | Not present | Present | Not present | Not present | Mild steatosis | *Present* | Not assessed | Present | Peribiliary lymphocytosis in right bx | HCC |
|  |  |  | At shunt closure, right lobe wedge right lobe | Slender bridging | Present (2/17) | 7/8/0 | Present | Increased/Normal | Not present | Present | Not present | Not present | Not present | *Present* | Not assessed | Present | Peribiliary lymphocytosis in right bx | HCC |
| **5** | Female/  14 years | 2 | Resection of lesion from left lobe (too little non-lesional tissue). Core needle biopsy from right | None | Not present | 2/6/3 | Not present | Normal/Normal | Not present | Not present | Present | Not present | Not present | Present | Not present | Not present | Normal | FNH |
| **6** | Male/  2 years | 2 | At shunt closure. Wedge biopsy | Mild periportaand perisinusoidal | Present (1/5) | 0/4/0 | Not present | Normal/Normal | Not present | Not present | Present | Not present | Not present | Not present | Not present | Not present | Normal | Not present |
| **7** | Female/  2 years | 2 | At shunt closure, left lobe wedge | Slender bridging | Present (7/13) | 0/4/1 | Not present | Increased/Focally large | Present | Present | Present | Not present | Not present | Present | Not present | Not present | Normal | Not present |
|  |  |  | At shunt closure, right lobe wedge | Slender bridging | Present(10/51) | 1/32/8 | Not present | Increased/Focally large | Present | Present | Present | Not present | Not present | Present | Not present | Not present | Normal | Not present |
| **8** | Female/  5 years | 2 | At shunt closure wedge biopsy | Slender bridging | Present(14/40) | 13/9/4  One portal vein with thickened wall. | Present | Increased/Normal | Present | Present | Present | Not present | Not present | Not present | Not present | Present | Peribiliary lymphocytosis | Not present |
| **9** | Male/  16 years | 2 | At shunt closure, left lobe wedge | Mild periportal fibrosis | Present (27/35) | 6/2/0 | Present | Increased/Focally large | Present | Not present | Not present | Not present | Present (moderate) | Present | Present | Not present | Normal | Not present |
|  |  |  | At shunt closure, right lobe wedge | Mild periportal fibrosis | Present (42/81) | 16/13/0 | Present | Increased/Focally large | Present | Not present | Not present | Not present | Present (moderate) | Present | Present | Not present | Normal | Not present |
| **10** | Male/  2 years | 2 | At shunt closure wedge biopsy | None | **Not present (0/17)** | **1/16/0** | Not present | Increased/Normal | Present | Present | Present | Not present | Not present | Present | Not present | Not present | Peribiliary lymphocytosis | Not present |
| **11** | Male/  7 years | 2 | Core needle biopsy right lobe in 2014 at time of repeating shunt due to failure of previous closure | Slender bridging | Present (1/6) | 0/5/0 | Present | Normal/Normal | Not present | Present | Present | Not present | Not present | Not present | Not present | Not present | Normal | FNH |
| **12** | Male/  12 years | 1 | Core needle biopsy 1 month before shunt closure | Mild perisinusoidal | **Not present (0/2)** | **0/2/0** | Not present | Increased/Normal | Not present | Present | Present | Not present | Not present | Present | Present | Not present | Not present | FNH |
| **13** | Female/  9 months | 2 | Right lobe wedge at time of shunt closure 3 years old | None | Present (29/43) | 0/13/0 | Not present | Increased/Normal | Present | Present | Present | Not present | Not present | Not present | Not present | Not present | Normal |  |
|  |  |  | Wedge biopsy taken at second stage closure 5 year old | None | Present (42/48) | 0/6/0 | Present | Increased/Normal | Present | Present | Present | Not present | Present, mild | Present | Not present | Not present | Normal |  |
| **14** | Female/  2 months | Intrahepatic | First core needle biopsy at 2 months | Mild periportal and perisinusoidal | Present (2/3) | 0/1/0 | Not present | Increased/Normal | Present | Present | Not present | Not present | Not present | *Present* | Not present | Not present | Normal | Not present |
|  |  |  | Second core needle biopsy taken at 6 years at the time of hepatic venogram | Mild periportal | Not present (0/8) | **4/0/4** | Not present | Increased/Normal | Present | **Not assessed** | Not present | Not present | Not present | Present | Not present | Not present | Normal |  |
| **15** | Male/  8 years | 2 | Core needle biopsy at 8 years | Mild periportal fibrosis | Not present (0/6) | 3/0/3 | Present | Normal/Normal | Present | Not present | Present | Not present | Not present | Present | Not present | Not present | Normal | FNH |
|  |  |  | core biopsy at time of shunt closure 3 months later | Mild periportal fibrosis | **Present (1/12)** | **5/0/6.** | Not present | Increased/Normal | Present | Present | Present | Not present | Not present | Not present | Not present | Present | Normal |  |
| **16** | Female/  2 months | Intrahepatic | Core needle biopsy at 2 months.  Spontaneous closure at 1 year | Mild periportal and perisinusoidal | **Not present (0/2)** | **1/1/0** | Present | Increased/Normal | Present | Not present | Not present | Not present (giant cells) | Not present | *Present* | Present | Not present | Normal | Not present |
| **17** | Male/  4 months | 2 | Wedge biopsy at shunt closure at 2 years | Slender bridging | Present (8/9) | 0/1/0 | Present | Increased/Normal | Present | Not assessed | Present | Not present | Not present | Not present | **Not assessed** | Not present | Peribiliary lymphocytosis | FNH |
| **18** | Female/  17 years | 1 | Wedge biopsy at second stage closure | Mild portal and perisinusoidal | Present (1/13) | 0/9/3 | Not present | Increased/Normal | Not present | Present | Present | Not present | Not present | Not present | Not present | Not present | Normal | Not present |
| **19** | Female/  2 years | 2 | First core needle biopsy at two years | Mild periportal | **Present (1/3)** | **1/0/1** | Not present | Normal/Normal | Present | Not present | Not present | Not present | Not present | Not present | **Not assessed** | Present (focal) | Normal | Not present |
|  |  |  | Wedge biopsy at shunt closure at 25 years | Mild periportal fibrosis | Present (20/22) | 2/0/0 | Not present | Increased/Focally large | Not present | Not present | Not present | Not present | Not present | Not present | Not present | Not present | Normal |  |
| **20** | Male/  49 years | 1 | Core needle biopsy at time of diagnosis of HCC. Small sample | None | Present (1/5) | 1/2/1 | Not present | Increased/Normal | Present | Present | Not present | Present ( Cholestasis) | Present, moderate | Not present | Not present | Not present | Normal | HCC |
| **21** | Female/  6 years | 2 | Wedge biopsy at shunt closure | None | **Present (1/12)** | **0/4/7** | Not present | Normal/Normal | Not present | Present | Present | Not present | Not present | Present | Not present | Present (focal) | Normal | Not present |
| **22** | Male/  2 years | 2 | Wedge biopsy at the time of closure 5 months later | Slender bridging | Present (11/15) | 0/2/2 | Not present | Increased/Normal | Present | Present | Not present | Not present | Not present | Present | Present | Present (focal) | Normal | Not present |

The type of CPSS was subclassified into four types :

-extrahepatic end to side shunt with no detectible flow into the intrahepatic portal system, (type 1);

-extrahepatic either side to side or H type shunt with some preserved intrahepatic portal flow, (type 2);

-intrahepatic shunt(s), any configuration except for persistent ductus venosus (intrahepatic type);

-persistent ductus venosus (PDV)
